# Intraspecific scaling of the minimum metabolic cost of transport in leghorn chickens (*Gallus gallus domesticus*): links with limb kinematics, morphometrics and posture

**DOI:** 10.1242/jeb.111393

**Published:** 2015-04

**Authors:** Kayleigh A. Rose, Robert L. Nudds, Jonathan R. Codd

**Affiliations:** Faculty of Life Sciences, University of Manchester, Manchester M13 9PT, UK

**Keywords:** Terrestrial locomotion, Size, Body mass, Geometric similarity, Energetics

## Abstract

The minimum metabolic cost of transport (CoT_min_; J kg^−1^ m^−1^) scales negatively with increasing body mass (∝*M*_b_^−1/3^) across species from a wide range of taxa associated with marked differences in body plan. At the intraspecific level, or between closely related species, however, CoT_min_ does not always scale with *M*_b_. Similarity in physiology, dynamics of movement, skeletal geometry and posture between closely related individuals is thought to be responsible for this phenomenon, despite the fact that energetic, kinematic and morphometric data are rarely collected together. We examined the relationship between these integrated components of locomotion in leghorn chickens (*Gallus gallus domesticus*) selectively bred for large and bantam (miniature) varieties. Interspecific allometry predicts a CoT_min_ ∼16% greater in bantams compared with the larger variety. However, despite 38% and 23% differences in *M*_b_ and leg length, respectively, the two varieties shared an identical walking CoT_min_, independent of speed and equal to the allometric prediction derived from interspecific data for the larger variety. Furthermore, the two varieties moved with dynamic similarity and shared geometrically similar appendicular and axial skeletons. Hip height, however, did not scale geometrically and the smaller variety had more erect limbs, contrary to interspecific scaling trends. The lower than predicted CoT_min_ in bantams for their *M*_b_ was associated with both the more erect posture and a lower cost per stride (J kg^−1^ stride^−1^). Therefore, our findings are consistent with the notion that a more erect limb is associated with a lower CoT_min_ and with the previous assumption that similarity in skeletal shape, inherently linked to walking dynamics, is associated with similarity in CoT_min_.

## Abstract (precis)

**Summary:** Chickens with differing body size and posture but similar skeletal shape show no difference in the cost of transport.

## INTRODUCTION

Body size has a significant influence on the morphology and metabolism of animals (Schmidt-Nielsen, 1975, 1984; Biewener, 1989). In animals that locomote terrestrially, the absolute amount of metabolic energy required to move a given distance increases with increasing body size, but not in direct proportion (slope <1) (Bruinzeel et al., 1999; Halsey and White, 2012). In relative terms, the mass-specific energy per unit distance (the cost of transport, CoT; J kg^−1^ m^−1^) is lower in larger species than in smaller ones. Often, at optimal self-selected speeds within a gait, animals incur a minimum cost of transport (CoT_min_) and it seems reasonable to expect natural selection to favour strategies that minimise the CoT_min_. For example, if the movement requirements of animals were similar, they would be expected to share optimum limb dynamics, and similar morphological proportions to allow it (Alexander and Jayes, 1983). The evolutionary allometry of CoT_min_ with body mass (*M*_b_, kg) is widely reported. For example, across more than 90 species of mammals and birds (7 g to 260 kg), CoT_min_=10.7*M*_b_^−0.32^ (Taylor et al., 1982). Adding amphibians, reptiles and invertebrates (<1 g) to this data set yielded a similar result (CoT_min_=10.8*M*_b_^−0.32^; Full and Tu, 1991) and African elephants (*Loxodonta africana*, *M*_b_=1542 kg) fall within the 95% confidence intervals (CIs) of this equation (Langman et al., 1995). The scaling exponent, however, is known to differ between walking and running (Margaria et al., 1963; Minetti et al., 1999; Rubenson et al., 2004, 2007; Maloiy et al., 2009; Nudds et al., 2011; Watson et al., 2011), and also between small crouched- and large upright-postured vertebrates (Reilly et al., 2007; Nudds et al., 2009). Furthermore, there is overlooked variation in CoT_min_ at a given *M*_b_, associated with variation in body form (Full et al., 1990). The general trend of decreasing CoT_min_ with *M*_b_, however, holds for over three orders of magnitude. Where outliers exist, their relatively more or less economical CoT_min_ compared with other species of the same *M*_b_ is attributed to adaptations associated with activity patterns (Watson et al., 2011), dominant locomotor mode (Dawson and Taylor, 1973; Fish et al., 2000, 2001; Griffin and Kram, 2000; Nudds et al., 2010), ecological niche (Bruinzeel et al., 1999), climate (Yousef et al., 1989; Maloiy et al., 2009) or having a protective shell (Baudinette et al., 2000; Zani and Kram, 2008). Ultimately, the reasons underlying the allometry of CoT_min_ with *M*_b_ and the factors that determine the CoT are not yet fully understood (Cavagna et al., 1977; Fedak et al., 1982; Heglund et al., 1982a,b; Heglund and Taylor, 1988; Kram and Taylor, 1990; Roberts et al., 1998; Pontzer, 2005, 2007a,b).

Between disparate species, musculoskeletal morphology and shape vary with size (Schmidt-Nielsen, 1975, 1984; Biewener, 1989; Reilly et al., 2007), speed requirements (Garland, 1983), climate (Janis and Wilhelm, 1993), ecological niche (Bruinzeel et al., 1999) and locomotor mode (Griffin and Kram, 2000; Abourachid, 2001; Nudds et al., 2010). Within species or between closely related species, however, variation in shape is reduced, meaning insight can be gained into the factors that dictate the CoT and how it scales with *M*_b_ independent of shape (Griffin et al., 2004; Day and Jayne, 2007; Langman et al., 2012). For example, miniature, Arabian and draft horses (*Equus ferus caballus*) showed no difference in CoT_min_ when trotting, despite spanning 8- and 2-fold differences in *M*_b_ and leg length, respectively (Griffin et al., 2004). Similarly, there was little difference in walking CoT_min_ within camels (*Camelus dromedaries*, *M*_b_*=*240–580 kg) (Yousef et al., 1989; Maloiy et al., 2009) or donkeys (*Equus asinus*, *M*_b_*=*170–583 kg) (Yousef et al., 1972; Maloiy et al., 2009), or between adult Asian elephants (*Elephas maximus*) and sub-adult African elephants (*M*_b_*=*1435–3545 kg) (Langman et al., 1995, 2012). It is assumed that similarity in CoT_min_ across individuals of differing body masses is due to their being geometrically, posturally and physiologically similar and locomoting with dynamically similar gaits (Griffin et al., 2004; Langman et al., 2012). Surprisingly, despite this explanation being widespread in the literature, there is no empirical evidence linking CoT_min_ across a size range with similar limb kinematics and skeletal proportions for a walking gait (the only gait over which dynamic similarity can be investigated; Alexander and Jayes, 1983). In humans, the only bipedal species to have been examined across a size range (children–adults), walking CoT_min_ scaled in a similar manner to that found across species (i.e. ∝*M*_b_^−1/3^) (Weyand et al., 2010), which is contrary to findings from within quadruped investigations where CoT_min_ was similar across sizes. To fully understand these results, it is necessary to expand the available data for bipeds and to investigate the relationships between the CoT, *M*_b_, limb kinematics and skeletal proportions.

**List of symbols and abbreviations**

CoT_min_: minimum cost of transport
CoT_net_: net cost of transport
CoT_tot_: total cost of transport
*f*_stride_: stride frequency
*h*_hip_: hip height
*l*_skel_: skeletal leg length
*l*_stride_: stride length
*M*­_b_: body mass
net-*P*_met_: net metabolic power
*P*_met_: metabolic power
RMR: resting metabolic rate
*t*_stance_: stance duration
*t*_swing_: swing duration
*U*: speed
*V̇*_CO_2__: rate of carbon dioxide production
*V̇*_O_2__: rate of oxygen consumption

Domestic leghorn chickens, *Gallus gallus domesticus* (Linnaeus 1758), are selectively bred for large and bantam (miniature) varieties, providing an opportunity to investigate how size influences CoT_min_ independent of shape in an avian species. Rubenson et al. (2007) derived an interspecific scaling equation of walking CoT_min_ against *M*_b_ [CoT_min_=17.80(±2.98)*M*_b_^−0.471(±0.032)^] using minimum measured values of the net cost of transport (CoT_net_; the amount of energy required to move 1 kg over 1 m minus maintenance and postural costs) for a range of birds and mammals (0.29–1542 kg). The aim of this study was to investigate whether large (*N*=5; mean±s.e.m. *M*_b_=1.92±0.13 kg, range=1.62–2.19 kg) and bantam (*N*=9; *M*_b_=1.39±0.03 kg, range=1.29–1.54 kg) leghorns would show a 16% difference in CoT_min_ as predicted by the Rubenson et al. (2007) equation, and to compare their CoT_min_ with that of animals of a similar *M*_b_. Importantly, we simultaneously determined whether the two varieties of leghorn walked in a dynamically similar way and were geometrically and posturally similar to gain insight into the links between these integrated components of terrestrial locomotion.

## RESULTS

### Morphological measurements

Mean linear dimensions measured from large and bantam leghorns are presented in Table 1. The skeletal measurements of the bantams were, on average, ∼83% of those of the larger variety. Predicted hindlimb dimensions (Table 1) for the bantams, based on the percentage difference in sternum length between the two varieties, all fell within the range predicted from the large variety data (mean±s.e.m.), indicating that the axial and appendicular skeletons of the two varieties were geometrically similar. Independent samples *t*-tests (equal variances assumed unless otherwise stated) showed that, represented as a proportion of total skeletal leg length (*l*_skel_=femur+tibiotarsus+tarsometatarsus lengths), the femur (0.28 in both varieties) was not significantly different (equal variances not assumed: Levene's test, *F=*13.71, *P*=0.003) between varieties (*t*=1.00, d.f.=4, *P=*0.374). Similarly, the tibiotarsus (*t*=0.07, d.f.=12, *P*=0.948) and tarsometatarsus lengths (*t*=−1.26, d.f.=12, *P*=0.233) were the same proportion of total leg length in the two varieties (0.42 and 0.30, respectively). Femur width, as a proportion of femur length was also similar (*t*=1.63, d.f.=12, *P*=0.128) between the two varieties (0.11 and 0.10 in bantam and large leghorns, respectively). Similarly, the tibiotarsus width:length ratio (0.07 in both varieties) did not differ (equal variances not assumed: Levene's test, *F*=5.25, *P*=0.041) between varieties (*t*=1.07, d.f.=5.70, *P*=0.326) and nor did the tarsometatarsus width/length ratio, which was 0.10 in both (*t*=0.00; d.f.=12, *P*=1.00). The two varieties therefore shared similar hindlimb skeletal proportions.

**Table 1.**
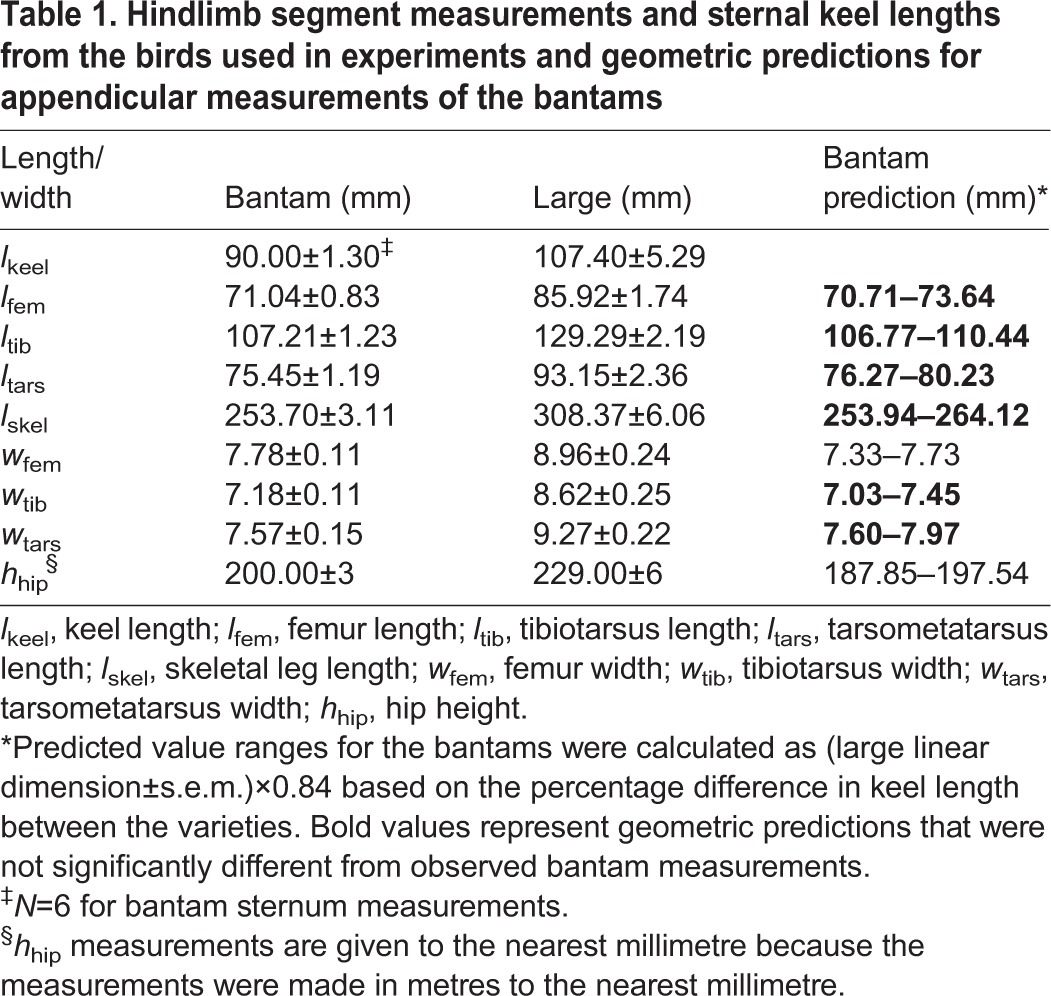
**Hindlimb segment measurements and sternal keel lengths from the birds used in experiments and geometric predictions for appendicular measurements of the bantams**

The ratio of hip height to skeletal leg length, *h*_hip_:*l*_skel_, a measure of posture (Gatesy and Biewener, 1991), was on average ∼5% greater in the bantam compared with the large variety (0.79±0.02 and 0.74±0.01, respectively), but was not statistically different between varieties (*t*=1.96, d.f.=12, *P*=0.074). The predicted *h*_hip_ for the bantams (Table 1), however, fell outside of the range predicted from the large variety’s *h*_hip_ data, being approximately 1 cm shorter than measured. Bantam *h*_hip_ was 0.87 times that of the larger birds, which was a greater fraction than found for the skeletal element measurements. Therefore, the bantams adopted a more erect posture compared with the large variety.

### Walking kinematics

**Fig. 1.**
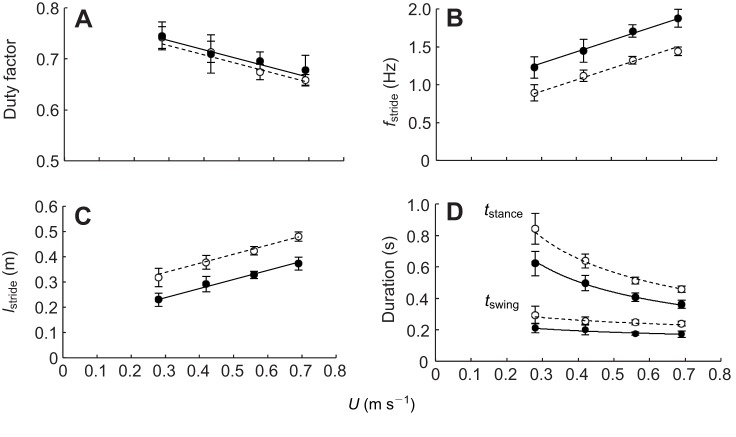
**Relationships between kinematics parameters and walking speed *U*.** Filled circles and solid lines represent data for bantam leghorns and open circles and dashed lines represent data for large leghorns. The lines of best fit are (A) duty factor=−0.18*U*+0.79 (bantam) and −0.18*U*+0.78 (large); (B) stride frequency, *f*_stride_=1.51*U*+0.83 (bantam) and 1.51*U*+0.46 (large); (C) stride length, *l*_stride_=0.36*U*+0.13 (bantam) and 0.36*U*+0.23 (large); and (D) swing time, *t*_swing_=0.16*U*^−0.22^ (bantam) and 0.21*U*^−0.22^ (large); and stance time, *t*_stance_=0.28*U*^−0.64^ (bantam) and 0.36*U*^−0.64^ (large). Data points are means±s.d. (s.e. are not large enough to be seen).

Duty factor decreased linearly with speed (*U*, m s^−1^) and neither the slope nor the intercept of this relationship differed between varieties (Fig. 1A, Table 2). Stride frequency (*f*_stride_, Hz) increased at the same rate with *U* in the two varieties, but was 0.37 Hz greater in the bantam variety across all *U* (Fig. 1B, Table 2). Similarly, the incremental increase in stride length (*l*_stride_, m) with *U* was the same in the two size groups, whilst *l*_stride_ was longer by 0.09 m across all *U* in the large variety (Fig. 1C, Table 2). The duration of the swing phase of the limb (*t*_swing_, s) decreased curvilinearly with *U* at the same rate in the two groups, but was 0.05 s longer in the large variety across all *U* (Fig. 1D, Table 2). Stance phase duration (*t*_stance_, s) also decreased curvilinearly with *U* and at the same rate in the two size groups. *t*_stance_ was, however, 0.08 s longer in the large variety across all *U* (Fig. 1D, Table 2). Therefore, each parameter responded to increasing *U* the same way in the two varieties and differences in their absolute values (related to size) were fixed across all speeds.

**Table 2.**
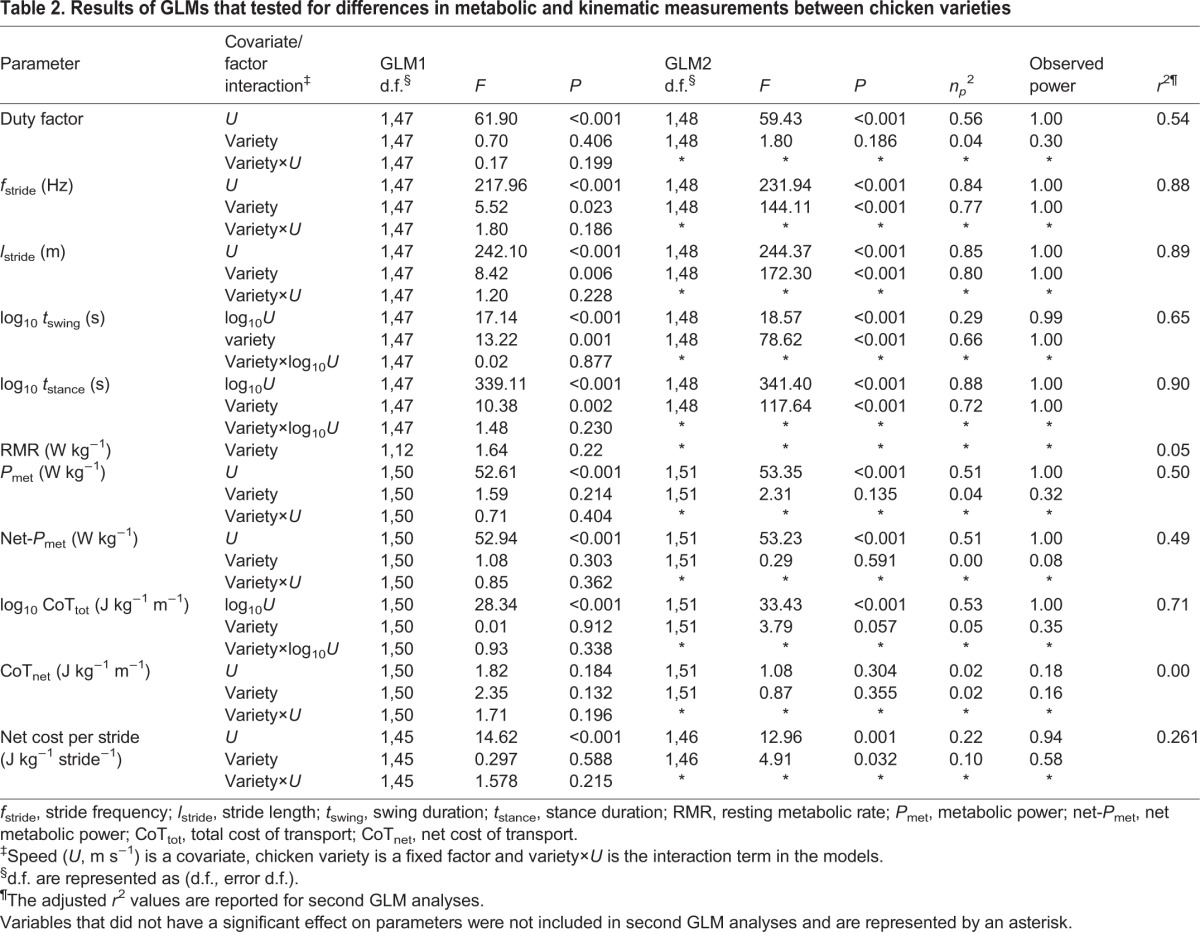
**Results of GLMs that tested for differences in metabolic and kinematic measurements between chicken varieties**

### Metabolic power and CoT

**Fig. 2.**
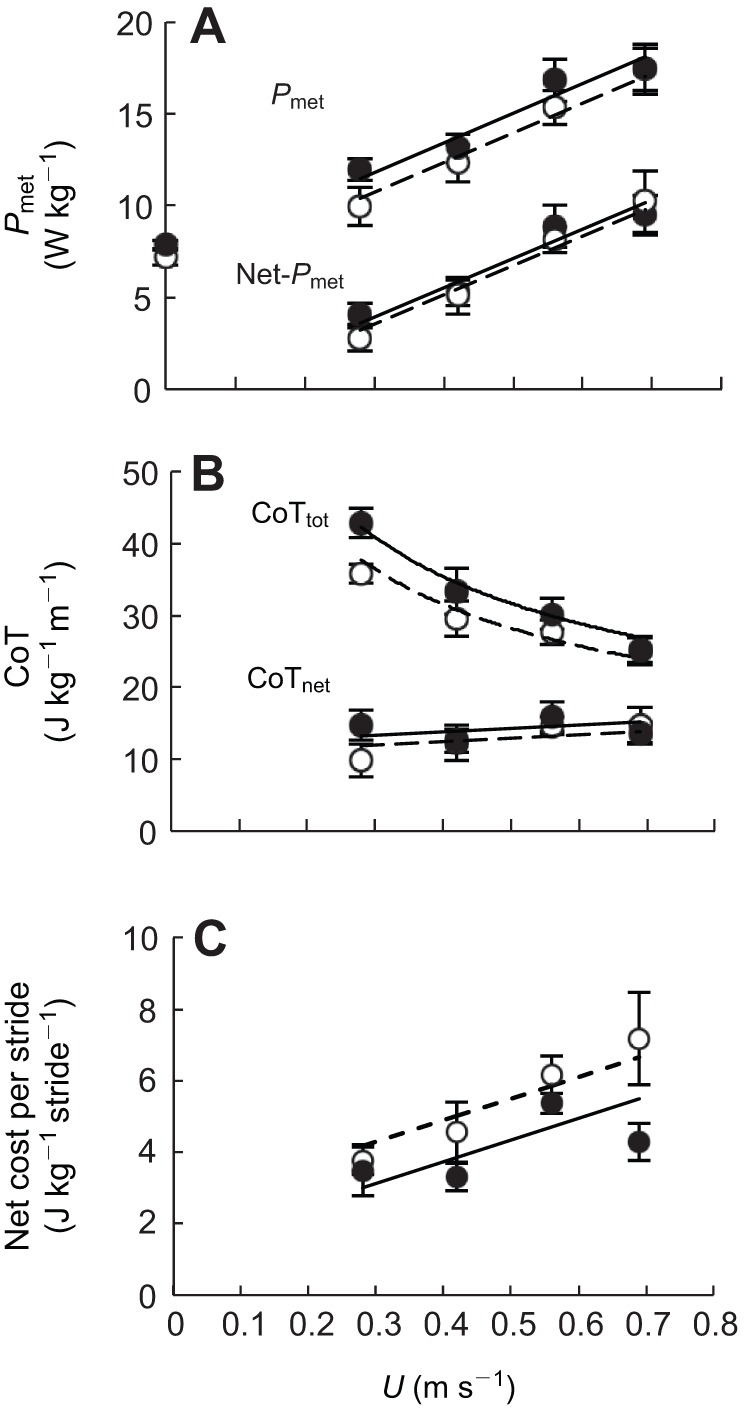
**Relationships between mass-specific energetic parameters and walking speed.** Data points and best-fit lines are as in Fig. 1. The lines of best fit are (A) metabolic power, *P*_met_=16.20*U*+6.93 (bantam) and 16.20*U*+5.86 (large); and net metabolic power, net-*P*_met_=16.00*U*−0.88 (bantam) and 16.00*U*−1.26 (large); (B) total cost of transport, CoT_tot_=22.39*U*^−0.50^ (bantam) and 19.95*U*^−0.50^ (large); and net cost of transport, CoT_net_=4.77*U*+11.89 (bantam) and 4.77*U*+10.53 (large); and (C) net cost per stride=7.10*U*+2.42 (bantam) and 21.21*U*+0.24 (large). Mass-specific resting (standing) metabolic rates are also included in A at 0 m s^−1^. Data points are means±s.e.m.

The positive relationship between mass-specific metabolic power (*P*_met_, W kg^−1^) and walking *U* (Fig. 2A) was similar (both the slopes and intercepts) for the two varieties (Table 2). Calculating CoT_min_ as the slope of this relationship (slope method) therefore gives 16.20 J kg^−1^ m^−1^ in each variety. During quiet standing, resting metabolic rate (RMR, W kg^−1^) did not differ (Fig. 2A, Table 2) between bantam and large leghorns (7.24±0.42 and 7.21±0.48 W kg^−1^, respectively), indicating that they shared the same mass-specific energetic cost of general maintenance and maintaining their posture combined. Therefore, the relationship between net mass-specific metabolic power (net-*P*_met_, W kg^−1^: the metabolic rate required for locomotion exceeding that required for standing quietly) and *U* (Fig. 2A) was also similar for the two size groups (Table 2).

Total cost of transport (CoT_tot_, J kg^−1^ m^−1^) decreased curvilinearly with *U*, indicating that the highest walking speeds of the birds were most metabolically optimal. CoT_net_ (J kg^−1^ m^−1^; net-*P*_met_/*U*), however, was not correlated with *U* and fell within a similar range for the two size groups (bantam: 9.44–16.10 J kg^−1^ m^−1^; large: 9.72–15.33 J kg^−1^ m^−1^) (Fig. 2B, Table 2). Calculating CoT_min_ as the minimum measured CoT_net_ (subtraction method), taken as the mean of all CoT_net_ values across all speeds and both varieties, gives 13.04 J kg^−1^ m^−1^. Predicted walking CoT_min_ values for large and bantam leghorns based on Rubenson et al. (2007) were 13.09 and 15.24 J kg^−1^ m^−1^, respectively. Both varieties therefore shared a CoT_min_ closer to that predicted for the larger variety, contrary to the 16% difference predicted. This corresponds to the bantams having a CoT_min_ ∼14% lower than predicted for their *M*_b_, which fell within the 95% CIs of Rubenson et al.’s (2007) equation. The net cost per stride (J kg^−1^ stride^−1^) was lower in bantams than in the larger variety by 1.17 J kg^−1^ stride^−1^ across all speeds (Fig. 2C, Table 2).

## DISCUSSION

Across species, CoT_min_ is reported to scale hypoallometrically with *M*_b_ (Taylor et al., 1970, 1982; Fedak et al., 1974; Kram and Taylor, 1990; Full and Tu, 1991; Langman et al., 1995; Roberts et al., 1998). However, we found that bantam and large varieties of leghorn chickens have identical CoT_min_ despite the smallest and largest individuals differing 1.7-fold in *M*_b_ and 1.35-fold in leg length. An independence of CoT_min_ from body size was previously reported within large quadrupedal species (>90 kg) spanning 1.5- to 8-fold ranges in *M*_b_ and up to 2-fold ranges in leg length (Griffin et al., 2004; Maloiy et al., 2009; Langman et al., 2012). The present data represent the first evidence of a lack of correlation between *M*_b_ and CoT_min_ within an avian species. No effect of *M*_b_ or leg length suggests that size itself does not influence the CoT but, rather, some other factor, perhaps correlated with body size, may be responsible.

The simultaneous collection of kinematics and morphological data here allow us to investigate further previous hypotheses on what is driving the interspecific CoT_min_ versus *M*_b_ relationship. Larger species perform the same amount of mass-specific mechanical work as smaller species, whilst using less mass-specific metabolic energy during terrestrial locomotion (Fedak et al., 1982; Heglund et al., 1982a,b; Alexander, 2005). How this is possible is not fully understood. It is generally accepted that *M*_b_ has no independent influence over CoT (Pontzer, 2005, 2007a,b). Leg length, however, is often discussed as the morphological factor explaining the allometry of CoT_min_ (Kram and Taylor, 1990; Schmidt, 1984; Biewener, 2003; Alexander, 2003) as longer legs allow longer *t*_stance_ for the muscles to apply force through recruiting slower, less metabolically expensive muscle fibres (metabolic rate is inversely proportional to *t*_stance_ during which the muscles apply force) (Kram and Taylor, 1990). In addition, longer limbs allow lower *f*_stride_, requiring fewer muscle contractions. In the present study, however, the different sized birds shared the same mass-specific CoT_min_, despite the bantams having shorter limbs, shorter *t*_stance_ and higher *f*_stide_ compared with the larger variety. Using the maximum height of the limb as a strut (effective limb length, *h*_hip_) as the indicator of size has been shown to better predict CoT_min_ across species (*h*_hip_, *r*^2^=0.98) than using the sum of the skeletal element lengths (*l*_skel_, *r*^2^=0.78) (Steudel and Beattie, 1995; Pontzer, 2007a). Over a small size scale of analysis, however, it has been demonstrated that between-individual differences in limb arrangement (e.g. limb excursion angle), the cost of swinging the limb and the coefficient of converting metabolic energy into muscle force ‘*k*’ (which were not measured in this study) prevent a clear relationship between *h*_hip_ and CoT_min_ (Pontzer, 2005, 2007b). In agreement with Pontzer's (2005, 2007b) findings, despite the greater absolute *h*_hip_ of the larger variety, compared with the bantams, they did not have a lower CoT_min_. It may be that variation in limb excursion angle (i.e. the difference in posture), rather than *h*_hip_, dominated variation in CoT_min_. Indeed, by using a model to predict the rate of force production associated with both supporting body weight and swinging the limb as a function of all of these parameters, Pontzer (2007a) found this was a better predictor of metabolic rate than contact time, limb length or *M*_b_ at both interspecific and intraspecific levels. Equally, the shared CoT_min_ of the two varieties may be due to their identical appendicular and axial skeletal geometry, consistent with previous assumptions in intraspecific analyses (Langman et al., 2012).

Another potential explanatory factor is limb posture (linked to effective limb length). Across vertebrates, the limb bone lengths scale positively and almost geometrically with *M*_b_, but become increasingly more aligned with one another and less crouched (Biewener, 1989). A prominent step-change exists in the scaling of both CoT_min_ and the mechanical cost of transport (*E*_mech_; J kg^−1^ m^−1^) across species associated with crouched postures in those <1 kg and upright postures in those >1 kg, making their efficiency of transport (CoT_min_/*E*_mech_) approximately 7% and 26%, respectively (Reilly et al., 2007; Nudds et al., 2009). Unlike larger species with a more upright posture, small crouched-postured (non-cursorial) species do not benefit from elastic energy savings or pendular mechanisms (Reilly et al., 2007). Furthermore, a more vertical limb decreases the muscular force required to support a unit of body weight and improves the mechanical advantage of the muscles (Biewener, 1989). The change in posture with increasing size means that muscle stress is nearly independent of *M*_b_ across species (rather than ∝*M*_b_^1/3^). Griffin et al. (2004) suggested that between closely related individuals, consistent limb posture might account for consistent CoT_min_ across a range of body sizes as muscle stress would in this case scale geometrically (∝*M*_b_^1/3^). The volume of active muscle would therefore increase with size and counter any metabolic savings associated with having longer legs (Griffin et al., 2004). However, in the present study the shared CoT_min_ of the chicken groups did not correspond to a similar posture. When comparing the posture of the two size groups as *h*_hip_:*l*_skel_, the limbs were 5% more erect in the variety selected for smaller size. The shared CoT_min_ in this case is perhaps better explained by the posture and lower cost per stride of the bantams. Across avian species, *h*_hip_ represents a greater proportion of *l*_skel_ with increasing *M*_b_ (Gatesy and Biewener, 1991). One potential explanation for why we found the opposite to what would be expected, as well as the lower cost per stride in the bantams, may be that the two varieties differ in their derived muscle properties or architecture as a result of selective breeding.

The kinematic data indicate that with *U*, the two varieties shared identical rates of change in all parameters, which would be expected to imply geometric, postural and dynamic similarity. Each kinematic parameter differed between the two varieties only by a fixed value across all speeds. The larger variety took longer strides by 9 cm, took less frequent strides by 0.37 Hz and had longer durations of both swing and stance phases of the limb by 0.05 and 0.08 s, respectively. At a given absolute *U*, duty factor is generally higher in larger species than in smaller ones (Gatesy and Biewener, 1991); however, the duty factors of the chickens were not significantly different between size groups. Similarly, a selection of felid species spanning a 46-fold range in *M*_b_ were found to use similar duty factors at a similar walking speed (Day and Jayne, 2007). For what was previously an expectation (Griffin et al., 2004; Maloiy et al., 2009; Langman et al., 2012), the present data offer the first empirical evidence of a link between identical walking CoT_min_ in individuals of differing size and similar limb dynamics and skeletal geometry. We can speculate that for a given skeletal shape, regardless of *M*_b_, walking CoT_min_ may be consistent. Some additional studies in which shape was controlled for also support this idea. For example, adding back loads up to 50% of *M*_b_ has a negligible effect on the CoT in quadrupedal rats, dogs and horses as well as bipedal humans, guinea fowl and other birds (Taylor et al., 1980; Ellerby and Marsh, 2006; Tickle et al., 2010, 2013). Furthermore, obese and thin humans of the same height (likely to be similar in skeletal proportions) show no difference in CoT_min_ (Browning et al., 2006).

In contrast to our findings, a comprehensive study of 48 humans spanning a 6-fold range in *M*_b_ and 1.5-fold range in height concluded that CoT_min_ was ∝*M*_b_^−1/3^ (Weyand et al., 2010). This result, however, may be associated with ontogenetic differences in shape, because the human subjects ranged from 5 to 32 years of age and the data were intentionally separated into four size groups to reduce individual variability (Weyand et al., 2010). Indeed, dividing the CoT by body height accounted for the observed differences between the human size groups. Therefore, at any given speed, all subjects incurred the same CoT to cover the same horizontal distance relative to their own body height (Weyand et al., 2010). Small (2 g) ghost crabs (*Ocypode quadrata*), one of the few invertebrate species examined, were found to have a higher CoT than larger ones (47 g), despite their similar appearance in shape (Tullis and Andrus, 2011). In the absence of detailed kinematic and morphometric measurements, however, it is not possible to conclude much from this result. It is, of course, possible that the link we found here between energetics, kinematics and skeletal morphometrics may not be characteristic of species with more than two legs.

### Conclusions

Leghorn chickens selectively bred for large and bantam varieties shared the same walking CoT_min_ despite a 1.70-fold difference in *M*_b_ and 1.35- fold difference in total leg length between the smallest and largest individuals. These data represent the first evidence of CoT_min_ being independent of *M*_b_ within a small crouched-postured bipedal species. Our findings also provide the first evidence (for what was previously only assumed) of a link between this and similar walking dynamics and skeletal geometry. In contrast to interspecific trends, however, *h*_hip_ did not scale geometrically between varieties and represented a greater proportion of total leg length in the bantam variety compared with the large variety. All birds shared a CoT_min_ closer to that predicted for the larger variety and the CoT_min_ of the bantams was approximately 14% lower than predicted from their *M*_b_. Our findings are therefore in agreement with the general consensus that for a given body size, CoT_min_ decreases with limb erectness. The lower than predicted CoT_min_ in the bantams was also associated with lower mass-specific energy requirements per stride, compared with the larger variety, which may be linked to differences in their posture and/or their derived muscle morphology/physiology. We emphasise the importance of intraspecific in addition to interspecific investigations as well as the combination of kinematics, morphometric and posture measurements towards gaining insight into the factors that dictate CoT.

## MATERIALS AND METHODS

### Study species

Adult (>16 week) male bantam (*N=*9; mean±s.e.m. *M*_b_=1.39±0.03 kg) and large (*N*=5; *M*_b_=1.92±0.13 kg) leghorn chickens were purchased from a local breeder and housed in the University of Manchester's animal unit. All housing was maintained on a 13 h:11 h light:dark cycle, at 18–22°C. Food and water were provided *ad libitum*, and the birds were not fasted prior to experiments. Birds were trained for 1 week to locomote on a motorised treadmill (T60 Tunturi^®^, Finland) prior to data collection. All experiments were carried out in accordance with the Animals (Scientific Procedures) Act 1986, were approved by the University of Manchester Ethics Committee and performed under a UK Home Office Project Licence held by J.R.C. (40/3549).

### Respirometry

An open flow respirometry system (all equipment Sable Systems International^®^, Las Vegas, NV, USA) was used to measure the birds' rates of oxygen consumption (*V̇*_O_2__, ml min^−1^) and carbon dioxide production (*V̇*_CO_2__, ml min^−1^). Perspex^®^ respirometry chambers were built (bantam: 66×46.5×48 cm, large: 97.5×53.5×48 cm) and mounted upon the treadmill. Air was pulled through the chambers using a FlowKit 500 at flow rates (FR) of 150 l min^−1^ (bantam) and 250 l min^−1^ (large). Excurrent airflow was sub-sampled (0.11 l min^−1^) for gas analysis. Water vapour pressure (WVP) was measured using an RH-300 water vapour analyser before the air was scrubbed of H_2_O with calcium chloride (2–6 mm granular, Merck, Darmstadt, Germany) and passed through a CO_2_ analyser (CA-10A). The dry air was scrubbed of CO_2_ using soda lime (2–5 mm granular, Sigma-Aldrich, Steinheim, Germany) and passed through a dual absolute and differential O_2_ analyser (Oxilla II). Ambient air (scrubbed of H_2_O and CO_2_ as before) was simultaneously passed through a second O_2_ channel on the Oxilla II at 0.11 l min^−1^ by a pump (SS-3) to enable calculation of differential O_2_ concentration (ΔO_2_). CO_2_ traces were base-lined to calculate differential CO_2_ concentration (ΔCO_2_). Voltage outputs were recorded using a UI2 interface and analysed using ExpeData^®^ v 1.1.15 software. The accuracy of the respirometry set up (±5%) across all speeds was determined using a N_2_ dilution test (Fedak et al., 1981). Primary flow rates (FR) were adjusted to dry-corrected flow rates (FR_c_), to account for the H_2_O scrubbed from air samples prior to gas measurements using:
(1)
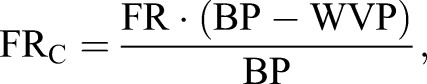
where BP is barometric pressure (measured with the Oxilla II) and WVP is water vapour pressure (Lighton, 2008). *V̇*_O_2__ was calculated using (Lighton, 2008):
(2)
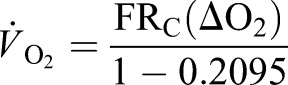
and *V̇*_O_2__ using (Lighton, 2008):
(3)
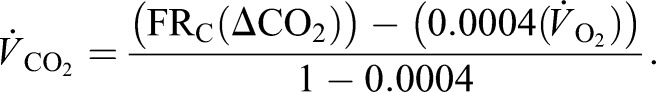


The birds were exercised over a range of randomised speeds (three per day) up to the maximum sustainable (bantam: 0.28–1.11 m s^−1^, large: 0.28–1.39 m s^−1^). Birds were given a rest of a minimum of 5 min to stand quietly between each period of exercise. RMRs were taken from the final rest period of each trial. Data were collected from stable gas readings lasting >1 min. Only data from speeds at which both varieties used a walking gait (0.28, 0.42, 0.56 and 0.69 m s^−1^) were included in analyses.

### Metabolic rate calculations

Five values were calculated at each speed: (1) *P*_met_ was converted from *V̇*_O_2__, using respiratory exchange ratios (RERs: *V̇*_O_2__:*V̇*_O_2__) and thermal equivalents taken from Brody (1945); (2) net-*P*_met_ was calculated by subtracting RMR from locomotor *P*_met_ (both from the same trial); (3) CoT_tot_ was calculated as *P*_met_/*U*; (4) CoT_net_ was calculated as net-*P*_met_/*U*; and (5) the cost per stride was calculated as net-*P*_met_/*f*_stride._

CoT_min_ was calculated using two methods: first, as the slope of the linear relationship between *P*_met_ and *U* (slope method) and, second, as the minimum measured CoT_net_ (subtraction method). CoT_min_ values calculated using the subtraction method were compared with predictions for walking birds and mammals of a similar *M*_b_ using eqn 3 from Rubenson et al. (2007).

### Gait kinematics

The birds were filmed (100 frames s^−1^) at all speeds in lateral view using a video camera (HDR-XR520VE, Sony, Japan). The left foot of each bird was tracked (∼10 strides) at each speed using Tracker software (v. 4.05, Open Source Physics) in order to quantify duty factor, *f*_stride_, *l*_stride_ (*U*/*f*_stride_), *t*_stance_ and *t*_swing_. Fluctuations in the kinetic and potential energy of the centre of mass (CoM) across a stride were determined through frame-by-frame tracking of a marker positioned over the left hip joint of the birds (indicative of *h*_hip_). To ensure that the birds were using a walking gait at all speeds analysed, the phase relationship between the horizontal kinetic energy (*E*_kh_) and the sum of the potential and vertical kinetic energies (*E*_p_+*E*_kv_) of the CoM (*h*_hip_) was determined. An out-of-phase relationship, indicating a walking gait, was found for all speeds used in the analyses.

### Morphological measurements

Keel length and the length and width (mid-shaft) of the right femur, tibiotarsus and tarsometatarsus was measured from the birds used in the respirometry experiments using digital vernier calipers (accuracy, ±0.01 mm). Geometric similarity in linear dimensions between the two size groups was investigated by determining whether their axial and appendicular dimensions scaled 1:1. The mean appendicular dimensions of the bantams were predicted based on the ratio of their keel length to that of the large variety. Skeletal element lengths were also compared as a percentage of total leg length. The ratio of *h*_hip_ to total skeletal leg length (*l*_skel_=femur+tibiotarsus+tarsometatarsus lengths) was calculated and used as a means of comparing posture between the two size groups, with a lower value indicating a more crouched posture. Back height (*h*_back_, m) was measured during the mid-stance as the distance from the hindtoe to the back at 90 deg to the direction of travel. Where birds (*N*=3) did not walk with ease with a hip marker, the ratio *h*_hip_:*h*_back_ (bantam: 0.80±0.01, large: 0.77±0.00) was used to estimate *h*_hip_.

### Statistical analyses

The slopes and the intercepts of the relationships between the dependent variables (metabolic or kinematics measures) and *U* were investigated for differences between chicken varieties using general linear models (GLMs). Models included variety as a fixed factor, *U* as a covariate and the interaction term variety×*U*. If the interaction term was non-significant (indicating similar slopes between varieties), it was removed from the model and the updated model was re-run (assuming parallel lines) in order to test for differences in intercepts. Where the relationship between a dependent variable and *U* was curvilinear, the data were log_10_ transformed. All best-fit lines were taken from coefficients tables produced by the GLMs. Between-variety differences in hindlimb skeletal element proportions (% total leg length) were investigated using independent samples *t*-tests. Hindlimb proportion data were tested for equality of variance using a Levene's test for equality of variance.
